# Supplementary material for: Analysis of PPARγ Signaling Activity in Psoriasis
Source: Int J Mol Sci. 2021 Aug 10;22(16):8603. doi: 10.3390/ijms22168603 (PMC8395241; doi:10.3390/ijms22168603)
Supplement: Supplementary file 1 [file ijms-22-08603-s001.zip › Supplemental materials_Analysis of PPARg signaling activity in psoriasis/Pathway models/Models images and html files/PPARG expressed in cells assotiated with psoriasis/115858.html]

subcutaneous fat


# Tissue subcutaneous fat

|  |  |
| --- | --- |
| URN | urn:agi-ncimtissue:C0222331 |
| Total Entities | 2 |
| Connectivity | 2497 |
| Name | subcutaneous fat |

---

|  |  |
| --- | --- |
| ChildConcepts | abdominal subcutaneous fat |
|  | subcutaneous white adipose tissue |

---

|  |  |
| --- | --- |
| Pathway | PPARG expressed in cells assotiated with psoriasis |

---

|  |  |
| --- | --- |
| MedScan ID | 6020174 |

---

|  |  |
| --- | --- |
| Alias | subcutaneous fat tissues |
|  | subcutaneous fats |
|  | subcutaneous adipose tissues |
|  | Subcutaneous fatty tissue |
|  | subcutaneous adipose tissue |
|  | sub-cutaneous adipose tissue |
|  | subcutaneous fat tissue |
|  | subcutaneous fat |
|  | Panniculus adiposus (Tela subcutanea) |
|  | Panniculus adiposus telae subcutaneae |
|  | Fatty layer of subcutaneous tissue |
|  | panniculus adiposus |
|  | Subcutaneous fatty tissues |

---

|  |  |
| --- | --- |
| NCIm ID | C0222331 |

---
